# Real-World Evaluation of Uromonitor^®^ for Bladder Cancer Detection and Surveillance

**DOI:** 10.3390/cancers18101650

**Published:** 2026-05-20

**Authors:** Amy Newman, Sasha Hansel, Gareth Gerrard, Llwyd Orton, Ashish Chandra, Rajesh Nair, Francesco Del Giudice, Youssef Ibrahim, Elsie Mensah, Muhammad Shamim Khan, Ramesh Thurairaja, Yasmin Abu Ghanem

**Affiliations:** 1 Cancer Genetics, Guy’s Hospital, Synnovis, London SE1 9RT, UK; 2 Guy’s Hospital, GSTT NHS Foundation Trust, London SE1 7EH, UK; 3 Neurophysiology, Manchester Metropolitan University, Manchester M15 6BX, UK; 4 Department of Maternal-Infant and Urological Sciences, “Sapienza” University of Rome, Umberto I Hospital, 00185 Rome, Italy

**Keywords:** non-muscle-invasive bladder cancer, surveillance, biomarker, TERT, FGFR3, KRAS, sensitivity, specificity

## Abstract

Background: Surveillance of non-muscle-invasive bladder cancer (NMIBC) relies on cystoscopy and urine cytology, both of which have well-recognised limitations. Molecular urine assays have been developed to reduce the burden of invasive surveillance, yet their real-world clinical utility remains uncertain. Uromonitor^®^ is a quantitative PCR-based assay targeting hotspot variants in the *TERT* promoter, *FGFR3*, and *KRAS*, which are frequently altered in urothelial carcinoma. We evaluated the performance of Uromonitor^®^ in routine clinical practice and assessed its technical reproducibility. Methods: Uromonitor^®^ diagnostic test accuracy was retrospectively calculated from samples from patients undergoing investigation for suspected bladder cancer (n = 64) or surveillance (n = 30) following a prior diagnosis at a tertiary referral centre between 2021 and 2023. Uromonitor^®^ results were compared with histology where available (n = 49, 52%), or with contemporaneous cystoscopy and urine cytology findings (n = 45, 48%). This pragmatic dual reference standard reflects routine clinical practice but may introduce some heterogeneity in diagnostic accuracy verification. A prospective in-house verification cohort was used to assess inter-laboratory reproducibility. Discordant cases underwent orthogonal next-generation sequencing (NGS) analysis. Sensitivity, specificity, positive predictive value (PPV), negative predictive value (NPV) and accuracy were calculated for the Uromonitor^®^ against the standard of care. Results: Ninety-four patients were included in the clinical performance analysis. Overall sensitivity, specificity, PPV, NPV and overall accuracy for Uromonitor^®^ were 38%, 88%, 63%, 72% and 70%, respectively. Sensitivity was higher in the diagnostic setting (47%; 95% CI 27.3–68.3%) than during surveillance (23%; 95% CI 8.2–50.2%). Several false-negative cases in the verification cohort harboured variants either detectable by NGS at variant allele frequencies below or slightly above the assay’s limit of detection or variants not covered by the assay hotspot design. Inter-laboratory reproducibility was excellent, with 100% concordance observed in the verification cohort. Conclusions: In a real-world clinical setting, Uromonitor^®^ demonstrated high specificity but limited sensitivity for detection of bladder cancer, particularly during surveillance. A negative result does not reliably exclude recurrence. Assay sensitivity thresholds and restricted variant coverage appear to be key contributors to false-negative results. These findings highlight the need for cautious clinical integration of Uromonitor^®^. It is unclear whether this approach has sufficient sensitivity in surveillance to safely reduce cystoscopy frequency. This underscores the need for further refinement of urine-based molecular assays, including a need for enhanced sensitivity and broader mutational coverage before routine clinical adoption.

## Simple Summary

Bladder cancer frequently recurs after initial treatment, requiring patients to undergo long-term monitoring. Current surveillance relies on cystoscopy, an invasive camera procedure, supported by urine cytology. Uromonitor^®^ is a urine-based genetic test targeting mutations in three genes, *TERT*, *FGFR3*, and *KRAS*, that are commonly altered in bladder cancer, offering a potential non-invasive alternative. We evaluated Uromonitor^®^ in 94 patients undergoing either initial investigation or routine surveillance at a UK tertiary centre. The test reliably identified true-positives (specificity ~88%) but missed a substantial proportion of cancers overall (sensitivity 38%) and performed particularly poorly during surveillance (sensitivity 23%), where the primary clinical need is to safely exclude recurrence. A negative result could not reliably rule out disease. Missed cases were largely explained by mutations below the test’s detection threshold or outside its target regions. While technically reproducible, Uromonitor^®^ cannot currently replace cystoscopy. Its potential role as an adjunct to cytology in selected patients warrants further prospective evaluation.

## 1. Introduction

Bladder cancer (predominantly urothelial transitional cell carcinoma) ranks among the most frequently diagnosed malignancies worldwide, imposing substantial clinical and economic burdens on healthcare systems. Approximately 75% of patients present with non-muscle-invasive bladder cancer (NMIBC), a biologically diverse disease marked by high recurrence rates and variable risk of progression to muscle-invasive disease [1,2]. Consequently, patients require long-term and often lifelong surveillance.

Current European Association of Urology (EAU) guidelines recommend cystoscopy as the cornerstone of NMIBC follow-up, supported by urine cytology, particularly for high-grade disease [3]. Whilst cystoscopy offers high sensitivity, it is invasive, costly, and uncomfortable for patients. Urine cytology, though non-invasive and highly specific, performs poorly in detecting low-grade tumours. These limitations have fuelled ongoing interest in urine-based biomarkers as potential adjuncts or alternatives to standard surveillance approaches.

Several urine biomarkers including NMP22 and UroVysion have gained FDA regulatory approval and ADX Bladder NICE approval. Despite this, none have been widely adopted in routine clinical practice or incorporated into major guidelines. Reported sensitivities have been inconsistent, especially for low-grade disease, and robust evidence demonstrating clear clinical benefit remains limited [4,5,6]. This uncertainty is reflected in expert consensus: 62% of specialists surveyed at the 2022 International Society of Urological Pathology Conference on Bladder Cancer agreed that insufficient evidence exists to support routine clinical use of molecular urine markers [7].

DNA-based urine assays represent a biologically plausible approach to bladder cancer detection, capitalising on the frequent shedding of tumour cells and tumour-derived DNA into urine. Uromonitor^®^ is a quantitative polymerase chain reaction (qPCR) assay targeting activating hotspot variants in the *TERT* promoter, *FGFR3*, and *KRAS* that rank among the most common genomic events in urothelial carcinoma. *TERT* promoter variants occur in approximately 60–80% of urothelial carcinomas across grades, whilst *FGFR3* activating variants are found in 40–60% of low-grade NMIBC and less commonly in high-grade or muscle-invasive disease. *KRAS* variants occur in approximately 5–9% of NMIBC and are mutually exclusive with *FGFR3* variants [8,9,10]. This molecular distribution means the assay’s target coverage varies by tumour stage or grade and may in part explain differences in performance across clinical settings. Early clinical studies reported encouraging diagnostic performance and suggested Uromonitor^®^ could serve as a useful adjunct to cystoscopy and cytology [11,12,13].

However, more recent independent investigations have reported substantially lower than anticipated sensitivity when applied in routine clinical settings, raising concerns about generalisability and underscoring the importance of real-world validation [14,15]. In particular, independent real-world evaluation of Uromonitor^®^ in the surveillance setting, where the clinical need is greatest, and systematic investigation of the molecular basis of false-negative results remain limited.

Differences in tumour burden, variant allele frequency, assay detection thresholds, and restricted variant coverage likely contribute to the discrepancies observed across studies.

Consequently, the aim of this study was to assess the diagnostic performance of Uromonitor^®^ in routine clinical practice, encompassing both initial diagnostic evaluation and surveillance of patients with NMIBC at a single major healthcare institution in the UK. We also evaluated inter-laboratory reproducibility and explored the molecular basis of discordant results using orthogonal next-generation sequencing. Based on the existing literature, we anticipated that the assay would demonstrate higher specificity than sensitivity, with sensitivity likely to be particularly limited in the surveillance setting where tumour burden and variant allele frequencies tend to be lower.

## 2. Materials and Methods

### 2.1. Study Design and Ethics

This retrospective observational study assessed the diagnostic performance of the Uromonitor^®^ urine assay (Uromonitor, Travessa Cruzes do Monte, Maia, Porto, Portugal) in routine clinical practice. The study was conducted at Guy’s and St Thomas’ NHS Foundation Trust (London, UK) and included patients tested between 2021 and 2023. Uromonitor^®^ testing was offered to patients attending the haematuria clinic or bladder cancer surveillance clinic during the study period based on clinical availability of the assay, rather than by strict consecutive enrolment.

Ethical approval was obtained through the Guy’s Cancer Cohort (reference 23/NW.0105, approved 13 March 2025). This research was supported by the Synnovis Innovation Accelerator Fund.

### 2.2. Patient Selection and Clinical Cohorts

Of 107 patients who underwent Uromonitor^®^ testing during the study period, 13 were excluded: five due to incomplete clinical or diagnostic data and eight with upper-tract urothelial carcinoma. Therefore, the final study population comprised 94 patients for retrospective analysis.

Patients were eligible if a Uromonitor^®^ result was available alongside sufficient contemporaneous clinical information, including histology where performed and/or cystoscopy and urine cytology findings. Each patient was included only once. Patients were grouped into two retrospective clinical cohorts:

- Diagnostic cohort: patients investigated for haematuria or suspected bladder cancer without prior history of bladder cancer (n = 64).
- Surveillance cohort: patients undergoing routine follow-up after previous bladder cancer diagnosis (n = 30).

A third cohort was studied prospectively for technical verification:

- Verification cohort: consecutive patients (n = 20) attending the bladder cancer clinic with either suspected or previously diagnosed bladder cancer had paired urine samples for Uromonitor^®^ inter-laboratory reproducibility testing.

### 2.3. Reference Standard and Clinical Assessment

The reference standard was histological confirmation of urothelial carcinoma where transurethral resection of bladder tumour (TURBT) or biopsy was performed. In cases where no tissue sampling was undertaken (i.e., normal cystoscopy appearance or clinically insignificant findings not warranting resection), absence of malignancy was determined by the combination of cystoscopy findings and urine cytology results as documented in the clinical record. This pragmatic approach reflects standard clinical practice where histological confirmation is not always feasible or appropriate.

Urine cytology was reported using the Paris System for Reporting Urinary Cytology [16]. Histological grading and staging followed institutional practice using the WHO 2004/2016 classification systems [17] and TNM staging [18] as documented in original pathology reports issued by Fellowship of the Royal College of Pathologists (FRCPath)-qualified histopathologists and cytopathologists.

### 2.4. Urine Collection and Molecular Testing

A minimum of 50 mL of voided urine was collected prior to instrumentation or cystoscopy to avoid contamination from traumatic catheterisation. Then, 10 mL aliquots were immediately filtered through the Uromonitor^®^ urine filtering device (UROKIT1-PUF25, U-monitor, Porto, Portugal) according to the manufacturer’s instructions. For the diagnostic and surveillance cohorts, one filter was processed at the U-Monitor reference laboratory (Porto, Portugal) using Uromonitor^®^ V2 testing. For the prospective verification cohort, paired filters were processed independently using the Uromonitor^®^ assay including the 2 additional *FGFR3* hotspots at both the manufacturer’s laboratory and the local Cancer Genetics Laboratory (Synnovis, London, UK).

The local laboratory process followed the instructions included with the Uromonitor assay. DNA extraction was performed manually using the Uromonitor^®^ DNA extraction and preparation kit (UROKIT2-PADNP25, U-monitor, Porto, Portugal) following the manufacturer’s protocol. Quantitative real-time PCR was carried out using 0.5–25 ng/μL of extracted DNA using the Uromonitor^®^ Real-Time PCR kit (UROKIT3-PRTPCR25, U-monitor, Porto, Portugal) on a QuantStudio 5 Real-Time PCR System (Thermo Fisher Scientific, Waltham, MA, USA). A variant was deemed to be present when a Ct value of ≤45 was achieved in the mutant hotspot target following PCR amplification of each of the selected hotspot regions in *TERT*, *KRAS* and *FGFR3*. No amplification of the variant or a Ct of >45 was regarded as negative.

The Uromonitor^®^ V2 assay targets the following recurrent hotspot variants frequently observed in urothelial carcinoma [19,20] (human reference genome GCRh37/hg19) with a lift-over to human reference genome GCRh38/hg38:

- *TERT* promoter (NM_198253.3): c.-124C>T (chr5:1295228C>T) and c.-146C>T (chr5:1295250C>T), referred to herein as *TERT* promoter 124 and 146, respectively.
- *FGFR3* (NM_000142.5): c.742C>T, p.(R248C), c.746C>G, p.(S249C).
- Additional *FGFR3* (NM_000142.5) variants included in verification cohort c.1108G>T, p.(G370C), c.1118A>G, p.(Y373C).
- *KRAS* (NM_001985.5): codons 12, 13, and 61 (multiple variants).

The manufacturer’s reported analytical sensitivity (limit of detection) is 3.125% variant allele frequency (VAF) for *FGFR3* variants and 6.250% VAF for *TERT* promoter and *KRAS* variants [21]. Samples were classified and reported as (i) positive if variants were detected above the assay threshold with appropriate amplification controls, (ii) negative if no variants were detected with adequate internal control performance, or (iii) inconclusive if quality control criteria (DNA quantity, amplification efficiency, or control performance) were not met. Results were reported as positive, negative, or inconclusive, with specific variants identified for all positive samples [22].

### 2.5. Inter-Laboratory Verification and Orthogonal Sequencing

For the verification cohort, paired 10 mL urine aliquots were independently filtered and processed following identical protocols at both the U-Monitor reference laboratory (Porto, Portugal) and the Cancer Genetics Laboratory (Synnovis, London, UK). Identical DNA extraction, PCR amplification, and variant detection were performed, and identical thermal cycling conditions and analysis software were used to ensure protocol alignment. Concordance was defined as identical classification (positive/negative/inconclusive) between laboratories and identical variant calls for all positive samples.

To investigate potential false-negative gene variant results, orthogonal validation by targeted next-generation sequencing (NGS) was performed on five Uromonitor^®^-negative samples in which histologically confirmed bladder cancer was present. Genomic DNA libraries were prepared from previously extracted urine source DNA (30–80 ng input) using the OncoDEEP^®^ assay targeted hybrid-capture panel (OncoDNA, Gosselies, Belgium), which interrogates 638 cancer-associated genes including comprehensive coverage of the *TERT* promoter, *FGFR3*, and *KRAS*. Library preparation utilised Twist universal adapters (Twist Bioscience, South San Francisco, CA, USA) followed by hybridisation-based enrichment. Sequencing was performed on a NovaSeq 6000 platform (Illumina, San Diego, CA, USA) using an SP flow cell with a target mean coverage depth of 500× for regions of interest. This assay was selected as it has been validated and in routine use for the pan-cancer workflow with formalin-fixed paraffin-embedded tissue at the local Cancer Genetics Laboratory. However, the assay has not previously been validated for DNA extracted from urine.

Sequence alignment to the human reference genome (GRCh38/hg38) and variant calling were performed using the OncoDNA cloud-based bioinformatics pipeline. Single nucleotide variants (SNVs) and small insertions/deletions (indels) were called if present at ≥2% variant allele frequency with a minimum of 12 supporting reads and adequate strand balance. Variants were annotated and interpreted using the OncoKDM variant interpretation platform (OncoDNA). For this comparative analysis, only variants within genes covered by the Uromonitor^®^ assay (*TERT* promoter, *FGFR3*, and *KRAS*) were analysed. Additional oncogenic and likely oncogenic variants detected by OncoDEEP^®^ are listed in the Supplementary Material (Table S2).

### 2.6. Data Collection

Clinical and demographic data were extracted from the electronic health record (Epic Systems Corporation, Verona, WI, USA), including patient age, sex, smoking history, presenting symptoms, cystoscopy findings, histological grade and stage (using WHO 2004/2016 classification and TNM staging), and urine cytology results (classified according to the Paris System). Treatment details were not recorded as part of this study. Follow-up data ranging from 6 to 24 months were collected where available to identify subsequent disease recurrence or progression beyond the index assessment.

### 2.7. Statistical Analysis

Diagnostic performance metrics were calculated. Sensitivity was defined as the proportion of patients with confirmed bladder cancer who had a positive Uromonitor^®^ result. Specificity was defined as the proportion of patients without bladder cancer who had a negative Uromonitor^®^ result. Positive predictive value (PPV) was defined as the proportion of positive Uromonitor^®^ results associated with confirmed bladder cancer. Negative predictive value (NPV) was defined as the proportion of negative Uromonitor^®^ results in patients without bladder cancer. Accuracy was defined as the proportion of patients whose Uromonitor^®^ test correlated with the result from their SOC.

Inconclusive Uromonitor^®^ results (n = 4) were excluded from the primary diagnostic accuracy analysis but were reported descriptively. Ninety-five-percent confidence intervals for all diagnostic performance metrics were calculated using the Wilson score method. Metrics were calculated both overall and separately for the diagnostic and surveillance cohorts.

## 3. Results

### 3.1. Study Population and Cohort Characteristics

Of the final study population, 64/94 patients (68%) were evaluated within the haematuria diagnostic pathway and had no prior history of bladder cancer. The remaining 30 patients (32%) were undergoing routine surveillance following previous bladder cancer diagnosis. Patient demographics showed a median age of 69 years (interquartile range [IQR]: 62–76 years), with 61 patients (65%) being male (Table 1).

Across both the diagnostic and surveillance cohorts, 32 patients (34%) had histologically confirmed bladder cancer at the time of Uromonitor^®^ testing. Tumour classification using the WHO 2004/2016 grading system identified five tumours as low-grade (16%) and 27 as high-grade (84%). Tumour staging among NMIBC cases showed 12 tumours classified as Ta (38%) and 16 as T1 (50%). Four patients had muscle-invasive disease (T2 or higher) (13%) (Table 2). Concomitant carcinoma in situ was present in eight cases (25% of malignant cases).

Overall, Uromonitor^®^ results were positive in 19 patients (20%), negative in 71 (76%), and inconclusive in 4 (4%). Among the 32 patients with confirmed bladder cancer, 12 (38%) had positive Uromonitor^®^ results and 20 (63%) had negative results (Table 2).

### 3.2. Diagnostic Cohort

Among the 64 patients in the diagnostic cohort, 45 (70%) had no evidence of bladder cancer following diagnostic work-up. Of these, 37 patients (82%) had a negative Uromonitor^®^ result (Table 2). Five patients (11%) had a positive Uromonitor^®^ result, all of which identified the same *FGFR3* R248C hotspot variant despite absence of malignancy on histology, cytology, and cystoscopy, suggesting a non-random pattern. These five false-positive patients remained cancer-free on follow-up cystoscopy at a median of 12 months (range: 6–18 months). Three patients (7%) had inconclusive Uromonitor^®^ results.

Nineteen patients (30%) in this cohort were diagnosed with bladder cancer on histology, including one low-grade tumour and 18 high-grade tumours. Uromonitor^®^ detected gene variants in nine of these patients (47%), whilst ten patients (53%) with histologically confirmed bladder cancer had negative Uromonitor^®^ results. Among the nine Uromonitor^®^-positive cases, three harboured *FGFR3* variants (two R248C and one S249C), four had a *TERT* promoter variant (one 124 and three 146), and two patients had concomitant *FGFR3* S249C and *TERT* 146 promoter variants (Supplementary Material Table S1).

Two patients with high-grade T1 disease harboured concomitant *FGFR3* S249C and *TERT* promoter variants at diagnosis. One patient subsequently underwent cystectomy due to high-risk disease, whilst the other has remained recurrence-free under surveillance over three years.

Among patients with negative Uromonitor^®^ results but confirmed malignancy (n = 20), urine cytology was available for 15 cases. Cytology results were negative in eight cases (53%), suspicious or atypical in five cases (33%), and high-grade malignant cells were identified in two cases (13%).

In the diagnostic cohort, Uromonitor^®^ demonstrated a sensitivity of 47% (95% CI 27.3–68.3%) and a specificity of 88% (95% CI 75.0–94.8%) for detection of bladder cancer. The PPV was 64% (95% CI 38.8–83.7%) and the NPV was 79% (65.1–88.0%), giving an overall diagnostic test accuracy of 75% (95% CI 63.3–84.5%) (Table 3).

### 3.3. Surveillance Cohort

The surveillance cohort comprised 30 patients with a prior bladder cancer diagnosis. Seventeen patients (57%) had no recurrence detected on cystoscopy at the time of testing (Table 3). Among these, 14 patients (82%) had a negative Uromonitor^®^ result, whilst two patients (12%) tested positive, one with an *FGFR3* S249C variant and the other with a *TERT* promoter 124 variant. One of these false-positive patients (*FGFR3* S249C) also had an atypical cytology result and 6 months later was diagnosed with prostate cancer but remained recurrence-free for bladder cancer at 24-month follow-up. The other remained disease-free at 12-month follow-up.

Thirteen patients (43%) in the surveillance cohort had recurrent disease confirmed by histology. Of these, three patients (23%) had a positive Uromonitor^®^ result (one R248C, one S249C and one *TERT* promoter 146), whilst ten patients (77%) had a negative result. Among the ten false-negative cases, seven had high-grade Ta or T1 disease, two had low-grade Ta disease, and one had T1 disease with concomitant carcinoma in situ.

Within the surveillance setting, Uromonitor^®^ demonstrated a sensitivity of 23% (95% CI 8.2–50.2%) and a specificity of 88% (95% CI 64.0–97.8%). The PPV was 60% (95% CI 23.1–92.1%), and the NPV was 58% (95% CI 40.7–74.5%), with an overall diagnostic test accuracy of 59% (95% CI 40.7–74.5%) (Table 3).

### 3.4. Overall Diagnostic Performance

Combining both the diagnostic and surveillance cohorts, 32 patients had confirmed bladder cancer at the time of testing. Uromonitor^®^ correctly identified 12 of these cases, yielding an overall sensitivity of 38% (95% CI 22.9–54.8%). Of the 62 patients without bladder cancer, 51 had negative Uromonitor^®^ results, yielding a specificity of 88% (95% CI 77.1–94.0%). The overall PPV was 63% (95% CI 41.0–80.9%), and the NPV was 72% (95% CI 60.5–81.0%), with an overall diagnostic test accuracy of 70% (95% CI 59.9–78.5%) (Table 3).

No *KRAS* variants were detected in any patient across all three cohorts. The most frequently detected gene variant was *FGFR3* R248C (n = 8 positive results, 3 true-positives), followed by *FGFR3* S249C (n = 5, all associated with malignancy), *TERT* promoter 124 (n = 2, 1 true-positive and 1 false-positive) and *TERT* promoter 146 (n = 6, all associated with malignancy).

### 3.5. Prospective Verification Cohort

Twenty urine samples were included in the prospective inter-laboratory verification cohort. All samples tested at the U-Monitor reference laboratory were reproduced locally at the Cancer Genetics Laboratory, demonstrating 100% concordance for both test classification and specific gene variant calls.

Two patients (10%) had detectable gene variants: one patient with prior history of muscle-invasive bladder cancer had a *TERT* promoter 124 variant detected, whilst one patient with newly diagnosed muscle-invasive disease had an *FGFR3* G370C variant identified. These patients underwent targeted NGS analysis using the OncoDEEP^®^ panel, the results of which were concordant; however, patient C11 had an additional *TERT* promoter 124 variant detected by OncoDEEP^®^ with a VAF of 7.42% (Table 4).

The remaining 18 samples (90%) were identified as negative by Uromonitor^®^.

Of the 18 Uromonitor^®^-negative samples, 13 patients (72%) had no evidence of recurrence or had suspicious cystoscopy findings subsequently confirmed as negative on histology. Two of these samples (patients C08 and C22) were randomly selected to undergo targeted NGS analysis using the OncoDEEP^®^ panel. The findings were concordant, with no *TERT* promoter, *FGFR3* or *KRAS* variants detected by either assay. No other oncogenic/likely oncogenic variants were detected (Supplementary Material, Table S2).

Five patients (28%), however, had new or recurrent NMIBC: four with newly diagnosed disease (three high-grade Ta and one high-grade T1) and one with recurrent low-grade Ta disease (Table 4).

### 3.6. Orthogonal NGS Analysis of False-Negative Cases

The five Uromonitor^®^-negative samples from the verification cohort with confirmed bladder cancer underwent targeted NGS analysis using the OncoDEEP^®^ assay panel. In one patient with recurrent low-grade disease, NGS analysis could not be completed due to insufficient sequencing coverage (mean depth <100×), likely reflecting low cell content in the urine sample.

Three samples harboured *TERT* promoter variants at variant allele frequencies (VAFs) ranging from 2.79% to 3.74%, below the reported detection threshold of 6.25% for the Uromonitor^®^ assay. The third sample (patient C20) had a *TERT* promoter 124 variant at 2.79% with a concomitant *FGFR3* Y373C at 8.25%. This VAF is higher than the LOD of 3.25% but was not detected by the Uromonitor^®^ This VAF is higher than the Uromonitor^®^ assay LOD of 3.125% for *FGFR* variants.

One case harboured an *FGFR3* S371C variant at a variant allele frequency of 38.95%, but since this was a hotspot not included in the Uromonitor^®^ assay’s target panel, it was not detected regardless of the relatively high VAF.

These findings demonstrate that both low variant allele frequency (below the 6.25% detection threshold for *TERT* promoter variants) and restricted hotspot coverage (*FGFR3* S371C not included in assay panel) contributed to false-negative Uromonitor^®^ results.

## 4. Discussion

### 4.1. Main Findings

In this real-world evaluation of the Uromonitor^®^ urine assay, we demonstrate that the test exhibits high specificity and excellent inter-laboratory concordance; however, its overall sensitivity for bladder cancer detection is limited, particularly in a surveillance setting. Our findings suggest that, in its current form, Uromonitor^®^ cannot reliably exclude bladder cancer when used as a stand-alone test. A strength of our approach is the integration of orthogonal NGS in discordant cases, which provides mechanistic insight into the causes of false-negative Uromonitor^®^ results. Our findings demonstrate insufficient analytical sensitivity to detect low-VAF mutations, and the restricted mutational coverage inherent in a qPCR approach contributes to the issue, highlighting the need for NGS-based approaches.

### 4.2. Comparison with Previous Studies

The observed diagnostic performance contrasts with several early validation studies reporting sensitivities exceeding 85–90% [11,12,13]. However, those studies were largely conducted in controlled or manufacturer-associated settings and often included selected patient populations. For example, Sieverink et al. reported a sensitivity of 93.1% and a specificity of 86.8% [12], whilst the SOLUSION trial by Azawi et al. demonstrated 89.7% sensitivity and 96.2% specificity [13]. In contrast, more recent independent investigations have reported substantially lower sensitivities when applied in routine clinical practice. Wolff et al. reported 49.3% sensitivity and 93.3% specificity in a large multicentre study [14], and Rubio-Briones et al. recently demonstrated 36% sensitivity and 93% specificity [15], values closely aligned with our findings of 38% overall sensitivity and 85% specificity. Our data therefore contribute to a growing body of evidence that assay performance in real-world clinical populations may differ considerably from early manufacturer-supported reports.

Importantly, we observed a clear difference in performance between diagnostic and surveillance contexts. Sensitivity was higher in the diagnostic cohort (47%) than among patients undergoing surveillance (23%). This may reflect differences in tumour burden, with newly diagnosed tumours shedding greater quantities of tumour-derived DNA into urine than small recurrent lesions. In the surveillance setting, where early detection of low-volume disease is most clinically valuable, the assay’s sensitivity was particularly limited. With an NPV of 58% in surveillance, nearly half of patients with negative molecular results still harboured recurrent disease. This finding has important clinical implications, as a false-negative result in this context may lead to inappropriate reassurance or delayed diagnosis.

### 4.3. Mechanisms Underlying False-Negatives

To explore the biological and technical basis of false-negative results, we performed orthogonal next-generation sequencing (NGS) of discordant cases. These findings indicate that both insufficient analytical sensitivity and restricted gene hotspot variant coverage contribute to missed detections. Had the assay possessed sufficient sensitivity to detect gene variants at 2–3% VAF, three of the five false-negative cases (60%) in the verification cohort would have been correctly identified, potentially improving sensitivity from 33% to 78% in that cohort. Taken together, these observations highlight a fundamental limitation of targeted PCR-based assays when applied to a biologically heterogeneous disease such as NMIBC. Patients C11 and C20 both had variants, *TERT* promoter 124 and *FGFR3* Y373C, respectively, at VAFs high enough to be detected by the Uromonitor^®^, but were not detected. One possible explanation for this may be the differences in calculation of measurements for sensitivity and LOD employed by qPCR and bioinformatic pipelines for NGS. qPCR measures the quantity of the target DNA following amplification [23], and NGS technology sequences individual DNA molecules [24]. Inefficiencies in primer binding, PCR inhibitors or amplification of artefacts may produce false-negative results [23].

Bladder cancer is characterised by substantial genomic diversity, with multiple driver alterations occurring across different grades and stages [19,20]. Whilst *TERT* promoter and *FGFR3* activating variants are common, they are not universal, and detectable variant allele frequency can vary widely depending on tumour size, grade, and cellular shedding. As demonstrated in this study, tumours harbouring relevant gene variants may still fall below the assay’s detection threshold, particularly in the surveillance setting, where tumour burden is typically lower. This limitation is not unique to Uromonitor^®^ and applies broadly to variant-specific urine assays relying on fixed analytical cut-offs.

### 4.4. Limitations of Mutation Coverage and Discordant Findings

Notably, no *KRAS* variants were detected in any patient across all three cohorts, despite *KRAS* being included as one of three target genes in the Uromonitor^®^ panel. This finding is consistent with other Uromonitor^®^ studies reporting very low *KRAS* variant detection rates [14,15] and reflects the relatively low frequency of *KRAS* variants in NMIBC, estimated at 5–9% in genomic sequencing studies [8,9,10]. The mutual exclusivity of *KRAS* and *FGFR3* variants in bladder cancer [10] may explain its inclusion in the assay design to capture *FGFR3*-wild-type tumours, but our data suggest limited added value for *KRAS* variant testing in this clinical setting.

False-positive results were observed in seven patients without contemporaneous evidence of bladder cancer, with five of these cases all harbouring the identical *FGFR3* R248C variants. This clustering may represent independent biological events and raises several possibilities, especially since *FGFR3* alterations have not been described in benign urothelial conditions. Potential explanations include: (1) field cancerisation with clonal patches of mutated urothelium below the threshold of endoscopic detection [25,26,27]; (2) low-level mosaicism in the urothelium; (3) preclinical disease that may manifest during longer follow-up; or (4) technical artefacts, though the latter seems less likely given the assay’s demonstrated reproducibility and absence of R248C contamination in negative controls. The median follow-up of 12 months (range 6–18) for these patients may be insufficient to exclude delayed cancer development. Longer-term follow-up data with repeat molecular testing would help clarify whether these represent true false-positives, field cancerisation, or early molecular detection preceding morphological changes.

The complementary role of urine cytology warrants consideration. In our cohort, cytology was available for 15 of 20 Uromonitor^®^-negative cases with confirmed malignancy. Notably, cytology was also negative in eight of these cases (53%), suspicious or atypical in five (33%), and positive for high-grade malignant cells in only two (13%). This suggests that neither test alone provides adequate sensitivity for surveillance, and that their combined use as currently recommended in guidelines [3] remains necessary. However, even the use of both approaches missed a substantial proportion of recurrences in our cohort, underscoring the continued need for cystoscopic surveillance as the reference standard.

Despite these limitations, the assay demonstrated excellent technical reproducibility, with complete concordance between the manufacturer’s reference laboratory and a tertiary molecular pathology laboratory. This finding confirms that assay performance variability is unlikely to be attributable to laboratory execution and instead reflects intrinsic assay characteristics. From a technical standpoint, Uromonitor^®^ can be reliably implemented within an established molecular diagnostics framework.

### 4.5. Clinical Implications and Future Directions

From a clinical perspective, the key question is whether the assay provides actionable information beyond existing standard-of-care tools. In our cohort, the limited sensitivity of 38% overall and particularly the 23% sensitivity during surveillance means that a negative Uromonitor^®^ result cannot safely replace or even reduce the frequency of cystoscopy. With a negative predictive value of only 58% in the surveillance setting, nearly half of patients with negative molecular results still harboured recurrent disease. Given the already substantial costs associated with long-term bladder cancer surveillance, estimated at $96,270–$172,426 per patient over 10–16 years in the United States [28,29,30], the introduction of additional tests without a demonstrable reduction in cystoscopy burden or improvement in clinical outcomes is difficult to justify. For clinical utility to be established, a urine-based test would need to demonstrate either: (1) sufficient sensitivity and negative predictive value to safely defer cystoscopy in low-risk scenarios, or (2) enhanced detection of high-risk disease that would alter management. Uromonitor^®^ in its current form does not meet these criteria.

Our findings must be considered in the context of other FDA-approved or CE-marked urine biomarkers. A systematic review by Laukhtina et al. reported pooled sensitivities for various urine tests ranging from 73% (Xpert Bladder Cancer Monitor) to 93% (CxBladder Monitor) for NMIBC surveillance, with specificities ranging from 76% to 84% [5]. More recent systematic reviews have reported median sensitivities of 65.7–100% for initial bladder cancer detection and 22.6–92% for surveillance across multiple platforms, including NMP22, BTA, UroVysion, ImmunoCyt, CxBladder, and Bladder EpiCheck [31,32]. However, these pooled estimates often derive from manufacturer-sponsored studies with selected populations, and substantial heterogeneity exists across studies. Real-world performance data similar to ours remain limited for most urine biomarkers, highlighting the need for independent validation studies across multiple platforms. The consistent observation of lower sensitivity in independent, real-world evaluations compared to early validation studies suggests this is a systematic issue affecting multiple biomarker technologies, not unique to Uromonitor^®^.

More comprehensive NGS-based assays offer higher sensitivity and broader gene variant coverage and were able to detect alterations missed by Uromonitor^®^ in this study. Here, we tested nine patients by OncoDEEP^®^ assay. Excluding one sample which failed sequencing metrics, the remaining eight samples showed 100% concordance when compared to the SOC. Recent targeted NGS panels for bladder cancer surveillance have demonstrated sensitivities exceeding 90% with appropriate depth of coverage and VAF thresholds [33,34]. However, such approaches remain expensive and resource-intensive, limiting their feasibility as routine surveillance tools at present.

Future directions for urine-based molecular surveillance should focus on addressing the limitations identified in this study. Potential strategies include: (1) lowering analytical detection thresholds to a sensitivity of 2–3% through improved PCR chemistry or digital PCR approaches; (2) expanding mutational coverage to include additional hotspots in *TERT* and *FGFR3* (including S371C) as well as other frequently altered genes (e.g., *PIK3CA*, *TP53*, and *KDM6A*); (3) developing personalised surveillance approaches where patient-specific variant panels are designed based on primary tumour sequencing; (4) investigating serial testing strategies to improve longitudinal sensitivity; and (5) exploring multimodal approaches combining DNA variant detection with epigenetic markers, RNA expression signatures, or protein biomarkers to enhance overall diagnostic performance. Importantly, any refined assay would require demonstration of clinical utility; specifically, the ability to safely reduce cystoscopy burden without compromising cancer detection before adoption into routine practice can be recommended.

### 4.6. Study Limitations

This study has several limitations that warrant consideration. First, the cohort size was modest (n = 94), and subgroup analyses by tumour grade and stage were underpowered, precluding definitive conclusions about test performance in specific risk categories. Second, discordant cases in the diagnostic and surveillance cohorts did not undergo orthogonal sequencing; comprehensive NGS analysis of all false-negative cases may have revealed additional mechanisms of test failure beyond those identified.

Of note, the inter-laboratory verification cohort comprised only 20 patients. While 100% concordance was observed, this sample size is too small to draw firm conclusions about reproducibility, and the finding should therefore be interpreted cautiously. Larger prospective studies would be needed to confirm these results. The OncoDEEP^®^ panel was selected because it is in routine validated use at our Cancer Genetics Laboratory for FFPE tissue and offers broad coverage of the relevant genes. However, it has not been formally validated for DNA derived from urine, which may affect the confidence of variant calls at low allele frequencies. NGS findings in false-negative cases should therefore be considered exploratory.

Third, longitudinal repeat testing was not performed, which may have clarified the temporal significance of isolated positive molecular results and whether serial testing improves diagnostic accuracy. Fourth, the retrospective design of the diagnostic and surveillance cohorts introduces potential selection bias, as Uromonitor^®^ testing may have been performed preferentially in certain clinical scenarios. Furthermore, Uromonitor^®^ testing was offered based on clinical availability of the assay rather than by strict consecutive enrolment, which may have introduced additional selection bias and limits the generalisability of the findings to all patients presenting to the haematuria or surveillance clinic during the study period.

Fifth, we did not compare Uromonitor^®^ directly with other commercially available urine biomarkers in head-to-head testing within the same cohort. Sixth, the relatively short median follow-up duration (12 months for false-positive cases, range 6–18 months) limits our ability to assess whether false-positive results represent preclinical disease or field cancerisation that may manifest over longer time periods. Seventh, cytology was not available for all patients, limiting our ability to comprehensively assess the complementary value of molecular testing and cytology. Finally, this was a single-centre study conducted within a specific healthcare system and population; external validation in diverse clinical settings, populations with different risk profiles, and healthcare systems with varying resources remains necessary. Nevertheless, our findings are concordant with other independent real-world evaluations [14,15] and provide important data on assay performance in routine practice, supporting the validity and generalisability of our conclusions.

## 5. Conclusions

Whilst Uromonitor^®^ demonstrated excellent technical reproducibility and high specificity, its limited sensitivity particularly in the surveillance setting precludes its use as a stand-alone test to safely defer cystoscopy. The assay’s performance is constrained by both insufficient analytical sensitivity to detect low-frequency variants and incomplete coverage of the gene variant landscape of bladder cancer. These limitations are shared by other variant-specific urine assays and highlight the challenges inherent in developing non-invasive tests for a genomically heterogeneous disease. Until urine-based biomarkers can demonstrate sufficient sensitivity, NPV, and clinical utility to meaningfully alter surveillance strategies, cystoscopy will remain the cornerstone of bladder cancer detection and monitoring. Our findings highlight the need for urine-based assays with both expanded molecular coverage and improved sensitivity.

## Figures and Tables

**Table 1:** Clinical data for diagnostic and surveillance cohorts.

Clinical Information	Characteristics	Diagnostic Cohort (n = 64)	Surveillance Cohort (n = 30)	Total Cases (n = 94)
Age	Median (years)	68	70	69
Gender	Male	36	25	61
	Female	28	5	33
TNM stage	Concomitant Cis	5	3	8
	Ta	6	19	25
	T1	1	18	19
	T2	2	3	5
Grade at diagnosis	No cancer	45	0	45
(WHO 2004/2016)	LG	1	12	13
	HG	18	18	36
Urine cytology	Total	61	20	81
	Positive/atypical	11	6	17
	Negative	49	14	63
	Suboptimal	1	0	1
Cystoscopy	Total	8	13	21
	Positive	7	12	19
	Negative	1	1	2
Presentation	Haematuria	41	12	53
	LUTS	5	0	5
	Other	14	18	32
	Asymptomatic	4	0	4

LUTS: lower-urinary-tract symptoms.

**Table 2:** Uromonitor results by grade (WHO 2004/2016) and stage (TNM), Cohorts A and B.

	Diagnostic Cohort					Surveillance Cohort					Combined	
Grade/Stage	Pos	Neg	INC	Total	Sensitivity (95% CI)	Pos	Neg	INC	Total	Sensitivity (95% CI)	Combined Total	Sensitivity (95% CI)
LG	0	1	0	1	0% (0–94.9%)	1	3	0	4	25% (1.3–69.9%)	5	20% (1.0–62.4%)
HG	9	9	0	18	50% (29.0–71.0%)	2	7	0	9	22% (3.9–54.7%)	27	40% (24.5–59.3%)
pTa	3	3	0	6	50% (18.8–81.2%)	2	4	0	6	33% (5.9–70.0%)	12	42% (19.3–68.0%)
pT1	5	6	0	11	45% (21.3–72.0%)	1	4	0	5	20% (1.0–62.4%)	16	38% (18.5–61.4%)
pT2	1	1	0	2	50% (2.6–97.4%)	0	2	0	2	0% (0–82.2%)	4	25% (1.3–69.9%)
No Cancer	5	37	3	45	N/A	2	14	1	17	N/A	62	N/A

Pos: positive; Neg: negative; INC: inconclusive; LG: low-grade; HG: high-grade.

**Table 3:** Sensitivity, specificity, positive predictive value (PPV), negative predictive value (NPV) and accuracy of the diagnostic, surveillance and prospective verification cohorts.

Metric	Diagnostic (95% CI)	Surveillance (95% CI)	Combined (95% CI)	Verification (95% CI)	All Cohorts (95% CI)
Sensitivity	47% (27.3–68.3%)	23% (8.2–50.2%)	38% (22.9–54.8%)	29% (50.8–64.1%)	36% (22.7–51.6%)
Specificity	88% (75.0–94.8%)	88% (64.0–97.8%)	88% (77.1–94.0%)	100% (77.2–100%)	90% (81.0–95.1%)
PPV	64% (38.8–83.7%)	60% (23.1–92.1%)	63% (41.0–80.9%)	100% (17.8–100%)	67% (45.4–82.8%)
NPV	79% (65.1–88.0%)	58% (38.8–75.5%)	72% (60.5–81.0%)	72% (49.1–87.5%)	72% (61.8–80.2%)
Accuracy	75% (63.3–84.5%)	59% (40.7–74.5%)	70% (59.9–78.5%)	75% (53.1–88.8%)	71% (61.8–78.6%)

PPV: positive predictive value; NPV: negative predictive value.

**Table 4:** Uromonitor and OncoDEEP mutation results for patients with a new diagnosis or recurrence of MIBC or NMIBC.

Patient	URM Result	OKD Result	OKD VAF%	Patient Stage at Index Assessment	Confirmed Diagnosis at Index Assessment
URM and SOC concordant					
C01	Positive (TERT 124)	Positive (TERT 124)	28%	Surveillance	HG pT2
C11	Positive (FGFR3 G370C)	Positive (FGFR3 G370C;	8.71%	New diagnosis	HG pT2
		TERT 124)	7.42%		
C08	Negative	Negative	—	Surveillance	No cancer detected
C22	Negative	Negative	—	Surveillance	No cancer detected
URM and SOC discordant					
C03	Negative	FAIL	—	Surveillance	LG pTa
C13	Negative	Positive (TERT 124)	3.58%	New diagnosis	LG pTa
C17	Negative	Positive (TERT 124)	3.74%	New diagnosis	Suspicious of HG (cytology)
C18	Negative	Positive (FGFR3 S371C)	38.95%	New diagnosis	HG pTa
C20	Negative	Positive (TERT 124;	2.79%	New diagnosis	LG pTa
		FGFR3 Y373C)	8.25%		

LG: low-grade; HG: high-grade; URM: Uromonitor; OKD: OncoDEEP assay; VAF: variant allele frequency.

## Data Availability

The data presented in this study are available from the corresponding author upon reasonable request.

## Supplementary Materials

The following supporting information can be downloaded at: https://www.mdpi.com/article/10.3390/cancers18101650/s1, Table S1: Bladder cancer grade, stage, cytology and Uromonitor^®^ result for individual patients: Cohort A (Diagnostic) and Cohort B (Surveillance); Table S2: Bladder cancer grade, stage, cytology, Uromonitor^®^ and OncoDEEP^®^ results for individual patients: Cohort C (Verification).
