# Supplementary material for: Real-World Evaluation of Uromonitor® for Bladder Cancer Detection and Surveillance
Source: Cancers (Basel). 2026 May 20;18(10):1650. doi: 10.3390/cancers18101650 (PMC13204559; doi:10.3390/cancers18101650)
Supplement: Supplementary file 1 [file cancers-18-01650-s001.zip › cancers-4273594-supplementary.pdf]

Supplementary Table 1. Bladder cancer grade, stage, cytology and Uromonitor® result for individual patients: Cohort A (Diagnostic) and Cohort B (Surveillance).

Patient	Sex	Presentation	Grade (WHO 1973)	Grade (WHO 2004)	Stage	Concomitant CIS	Cytology	URM Result	TERTp (NM_198253.3)	FGFR3 (NM_000142.5)	KRAS (NM_033360.4)	URM at Dx or Surveillance	Confirmed Tumour at URM
**DIAGNOSTIC COHORT (COHORT A)**													
1	Female	LUTS	N/A	N/A	N/A	N/A	Negative	Negative	-	-	-	Diagnosis	No
2	Female	Haematuria	N/A	N/A	N/A	N/A	Negative	Negative	-	-	-	Diagnosis	No
3	Male	Haematuria	G3	HG	pT1	No	Suspicious	Negative	-	-	-	Diagnosis	Yes
4	Female	Haematuria	G3	HG	pT1	Yes	Suspicious	Positive	c.-146C>T	c.746C>G	-	Diagnosis	Yes
5	Male	Other	N/A	N/A	N/A	N/A	Negative	Negative	-	-	-	Diagnosis	No
6	Female	Haematuria	N/A	N/A	N/A	N/A	Negative	Negative	-	-	-	Diagnosis	No
7	Male	LUTS	G3	HG	pT1	No	Negative	Negative	-	-	-	Diagnosis	Yes
8	Male	Haematuria	G3	HG	pT1	No	Negative	Positive	c.-146C>T	c.746C>G	-	Diagnosis	Yes
9	Female	Haematuria	N/A	N/A	N/A	N/A	Negative	Positive	-	c.742C>T	-	Diagnosis	No
10	Male	Haematuria	G3	HG	pT1	Yes	N/A	Positive	c.-146C>T	-	-	Diagnosis	Yes
11	Female	Haematuria	N/A	N/A	N/A	N/A	Negative	Negative	-	-	-	Diagnosis	No
12	Female	Haematuria	N/A	N/A	N/A	N/A	Negative	Negative	-	-	-	Diagnosis	No
13	Female	Haematuria	N/A	N/A	N/A	N/A	Negative	Negative	-	-	-	Diagnosis	No
14	Female	Haematuria	G3	HG	pT1	No	Negative	Negative	-	-	-	Diagnosis	Yes
15	Male	Haematuria	G3	HG	pT1	No	Suspicious	Negative	-	-	-	Diagnosis	Yes
16	Male	Other	N/A	N/A	N/A	N/A	N/A	Negative	-	-	-	Diagnosis	No
17	Female	Haematuria	N/A	N/A	N/A	N/A	Negative	Negative	-	-	-	Diagnosis	No
18	Female	LUTS	N/A	N/A	N/A	N/A	Negative	Negative	-	-	-	Diagnosis	No
19	Female	Haematuria	N/A	N/A	N/A	N/A	Negative	Negative	-	-	-	Diagnosis	No
20	Female	Other	N/A	N/A	N/A	N/A	Negative	Inconclusive	-	-	-	Diagnosis	No
21	Female	Asymptomatic	G2	LG	pTa	No	Negative	Negative	-	-	-	Diagnosis	Yes
22	Male	Other	N/A	N/A	N/A	N/A	Negative	Inconclusive	-	-	-	Diagnosis	No
23	Male	Haematuria	G3	HG	pT2	Yes	High grade	Positive	c.-146C>T	-	-	Diagnosis	Yes
24	Male	Haematuria	N/A	N/A	N/A	N/A	N/A	Negative	-	-	-	Diagnosis	No
25	Male	Other	N/A	N/A	N/A	N/A	Negative	Negative	-	-	-	Diagnosis	No
26	Male	LUTS	N/A	N/A	N/A	N/A	Negative	Negative	-	-	-	Diagnosis	No
27	Female	Asymptomatic	N/A	N/A	N/A	N/A	Negative	Negative	-	-	-	Diagnosis	No

Patient	Sex	Presentation	Grade (WHO 1973)	Grade (WHO 2004)	Stage	Concomitant CIS	Cytology	URM Result	TERTp (NM_198253.3)	FGFR3 (NM_000142.5)	KRAS (NM_033360.4)	URM at Dx or Surveillance	Confirmed Tumour at URM
28	Male	Haematuria	N/A	N/A	N/A	N/A	Negative	Negative	-	-	-	Diagnosis	No
29	Male	Haematuria	G2	HG	pTa	No	Suspicious	Positive	-	c.746C>G	-	Diagnosis	Yes
30	Male	Haematuria	N/A	N/A	N/A	N/A	Negative	Negative	-	-	-	Diagnosis	No
31	Female	Haematuria	G2	HG	pTa	No	Negative	Negative	-	-	-	Diagnosis	Yes
32	Male	Other	N/A	N/A	N/A	N/A	Negative	Negative	-	-	-	Diagnosis	No
33	Female	Haematuria	N/A	N/A	N/A	N/A	Negative	Positive	-	c.742C>T	-	Diagnosis	No
34	Female	Haematuria	G1	LG	pTa	No	Negative	Negative	-	-	-	Diagnosis	No
35	Female	Haematuria	N/A	N/A	N/A	N/A	Negative	Negative	-	-	-	Diagnosis	No
36	Male	Haematuria	N/A	N/A	N/A	N/A	Negative	Negative	-	-	-	Diagnosis	No
37	Male	Other	G2	HG	pT1	Yes	Suspicious	Negative	-	-	-	Diagnosis	Yes
38	Male	Haematuria	N/A	N/A	N/A	N/A	Negative	Negative	-	-	-	Diagnosis	No
39	Male	Haematuria	N/A	N/A	N/A	N/A	Negative	Negative	-	-	-	Diagnosis	No
40	Male	Haematuria	N/A	N/A	N/A	N/A	Negative	Negative	-	-	-	Diagnosis	No
41	Female	Haematuria	N/A	N/A	N/A	N/A	Negative	Inconclusive	-	-	-	Diagnosis	No
42	Male	Haematuria	N/A	N/A	N/A	N/A	Negative	Negative	-	-	-	Diagnosis	No
43	Female	Haematuria	N/A	N/A	N/A	N/A	Negative	Negative	-	-	-	Diagnosis	No
44	Female	Asymptomatic	N/A	HG	N/A	N/A	High grade	Negative	-	-	-	Diagnosis	Yes
45	Female	Haematuria	G3	HG	pT1	No	Negative	Positive	-	c.742C>T	-	Diagnosis	Yes
46	Male	Other	N/A	N/A	N/A	N/A	Negative	Negative	-	-	-	Diagnosis	No
47	Male	Other	-	N/A	-	-	Negative	Negative	-	-	-	Diagnosis	No
48	Male	Other	N/A	N/A	N/A	N/A	Negative	Negative	-	-	-	Diagnosis	No
49	Female	LUTS	N/A	N/A	N/A	N/A	Negative	Positive	-	c.742C>T	-	Diagnosis	No
50	Male	Haematuria	G3	HG	pT1	No	Suspicious	Positive	c.-146C>T	-	-	Diagnosis	Yes
51	Male	Haematuria	G2	HG	pTa	Yes	Negative	Positive	-	c.742C>T	-	Diagnosis	Yes
52	Female	Other	N/A	N/A	N/A	N/A	Negative	Negative	-	-	-	Diagnosis	No
53	Male	Haematuria	N/A	N/A	N/A	N/A	Negative	Negative	-	-	-	Diagnosis	No
54	Female	Asymptomatic	N/A	N/A	N/A	N/A	Negative	Positive	-	c.742C>T	-	Diagnosis	No
55	Male	Haematuria	G2	HG	pTa	N/A	High grade	Positive	c.-124C>T	-	-	Diagnosis	Yes
56	Male	Haematuria	G3	HG	pT2	No	Negative	Negative	-	-	-	Diagnosis	Yes
57	Male	Haematuria	N/A	N/A	N/A	N/A	Negative	Positive	-	c.742C>T	-	Diagnosis	No

Patient	Sex	Presentation	Grade (WHO 1973)	Grade (WHO 2004)	Stage	Concomitant CIS	Cytology	URM Result	TERTp (NM_198253.3)	FGFR3 (NM_000142.5)	KRAS (NM_033360.4)	URM at Dx or Surveillance	Confirmed Tumour at URM
58	Female	Haematuria	N/A	N/A	N/A	N/A	Negative	Negative	-	-	-	Diagnosis	No
59	Female	Haematuria	N/A	N/A	N/A	N/A	Atypical	Negative	-	-	-	Diagnosis	No
60	Male	Other	N/A	N/A	N/A	N/A	Suboptimal sample	Negative	-	-	-	Diagnosis	No
61	Male	Other	N/A	N/A	N/A	N/A	Negative	Negative	-	-	-	Diagnosis	No
62	Male	Other	G3	HG	pTa	No	Negative	Negative	-	-	-	Diagnosis	Yes
63	Male	Haematuria	N/A	N/A	N/A	N/A	Atypical	Negative	-	-	-	Diagnosis	No
64	Male	Haematuria	N/A	N/A	N/A	N/A	Negative	Negative	-	-	-	Diagnosis	No
**SURVEILLANCE COHORT (COHORT B)**													
1	Male	Haematuria	G1	LG	pTa	N/A	Negative	Positive	-	c.746C>G	-	Surveillance	Yes
2	Female	Other	G1	LG	pTa	No	N/A	Negative	-	-	-	Surveillance	Yes
3	Male	Other	G2	LG	pTa	No	N/A	Negative	-	-	-	Surveillance	Yes
4	Male	Other	G3	HG	pTa	N/A	Negative	Negative	-	-	-	Surveillance	No
5	Female	Haematuria	G3	HG	pTa	No	N/A	Negative	-	-	-	Surveillance	Yes
6	Male	Haematuria	G3	HG	pT1	Yes	Suspicious	Positive	-	c.742C>T	-	Surveillance	Yes
7	Female	Other	G2	HG	pT1	No	Negative	Negative	-	-	-	Surveillance	Yes
8	Female	Other	G3	HG	pTa	Yes	Negative	Negative	-	-	-	Surveillance	No
9	Male	Other	G2	LG	pTa	No	Atypical	Positive	-	c.746C>G	-	Surveillance	No
10	Male	Other	G1	LG	pTa	No	Negative	Negative	-	-	-	Surveillance	No
11	Male	Other	G2	HG	pT1	No	Negative	Negative	-	-	-	Surveillance	No
12	Male	Other	G3	HG	pT1	No	Negative	Negative	-	-	-	Surveillance	No
13	Male	Haematuria	G2	LG	pTa	No	Negative	Negative	-	-	-	Surveillance	No
14	Male	Haematuria	G3	HG	pTa	No	N/A	Negative	-	-	-	Surveillance	No
15	Male	Other	G3	HG	pT1	No	N/A	Negative	-	-	-	Surveillance	No
16	Male	Other	G2	LG	pTa	No	N/A	Negative	-	-	-	Surveillance	No
17	Male	Other	G2	HG	pTa	No	Atypical	Negative	-	-	-	Surveillance	No
18	Male	Other	G2	LG	pTa	No	N/A	Negative	-	-	-	Surveillance	No
19	Male	Other	G2	HG	pTa	No	N/A	Positive	c.-146C>T	-	-	Surveillance	Yes
20	Male	Haematuria	G3	HG	pT1	No	Negative	Negative	-	-	-	Surveillance	No
21	Male	Other	G3	HG	pT2	No	Negative	Negative	-	-	-	Surveillance	No

Patient	Sex	Presentation	Grade (WHO 1973)	Grade (WHO 2004)	Stage	Concomitant CIS	Cytology	URM Result	TERTp (NM_198253.3)	FGFR3 (NM_000142.5)	KRAS (NM_033360.4)	URM at Dx or Surveillance	Confirmed Tumour at URM
22	Male	Other	G3	HG	pT2	Yes	Atypia	Negative	-	-	-	Surveillance	Yes
23	Male	Other	G1	LG	pTa	No	N/A	Negative	-	-	-	Surveillance	Yes
24	Male	Other	G3	HG	pT1	No	High grade	Negative	-	-	-	Surveillance	Yes
25	Male	Haematuria	G2	LG	pTa	No	Negative	Negative	-	-	-	Surveillance	No
26	Female	Haematuria	G2	HG	pT1	No	Atypical	Negative	-	-	-	Surveillance	Yes
27	Male	Haematuria	G1	LG	pTa	No	Negative	Negative	-	-	-	Surveillance	No
28	Male	Haematuria	G1	LG	pTa	No	Negative	Positive	c.-124C>T	-	-	Surveillance	No
29	Male	Haematuria	G3	HG	pT1	No	Negative	Negative	-	-	-	Surveillance	Yes
30	Male	Haematuria	G3	HG	pT2	No	N/A	Negative	-	-	-	Surveillance	Yes

URM, Uromonitor® result; G, Grade (WHO 1973 classification); LG, low-grade (WHO 2004/2016); HG, high-grade (WHO 2004/2016); CIS, carcinoma in situ; TERTp, TERT promoter hotspot mutations (NM_198253.3); FGFR3, fibroblast growth factor receptor 3 (NM_000142.5); KRAS, Kirsten RAS proto-oncogene (NM_033360.4); Dx, first presentation/diagnosis; N/A, not applicable or information not available; –, no mutation detected. Patient numbers are sequential and independent within each cohort.

Supplementary Table 2. Bladder cancer grade, stage, cytology, Uromonitor® and OncoDEEP® results for individual patients: Cohort C (Verification).

Patient	Sex	Grade (WHO 1973)	Grade (WHO 2004)	Stage	Cytology	URM Result	TERTp (NM_198253.3)	FGFR3 (NM_000142.5)	KRAS (NM_033360.4)	URM at Dx or Surveillance	Confirmed Tumour/ Suspicious at URM	OKD Result	TERTp (OncoDEEP)	FGFR3 (OncoDEEP)	KRAS (OncoDEEP)
**VERIFICATION COHORT (COHORT C)**															
1	Male	G3	HG	pT2	N/A	Positive	c.-124C>T	-	-	Surveillance	Yes	Positive	c.-124C>T (27.7%)	-	-
2	Male	G3	HG	pTa	Negative	Negative	-	-	-	Surveillance	No	-	-	-	-
3	Male	G1	LG	pTa	N/A	Negative	-	-	-	Surveillance	Yes	Fail	Fail	Fail	Fail
4	Male	G1	LG	pTa	N/A	Negative	-	-	-	Surveillance	No	-	-	-	-
5	Female	N/A	N/A	N/A	N/A	Negative	-	-	-	Diagnosis	No	-	-	-	-
6	Female	N/A	N/A	N/A	N/A	Negative	-	-	-	Diagnosis	No	-	-	-	-
7	Male	G2	LG	pTa	N/A	Negative	-	-	-	Surveillance	No	-	-	-	-
8	Male	G3	HG	pT1	Negative	Negative	-	-	-	Surveillance	No	Negative	-	-	-
9	Male	N/A	N/A	N/A	N/A	Negative	-	-	-	Diagnosis	No	-	-	-	-
10	Male	G2	HG	pTa	Negative	Negative	-	-	-	Surveillance	No	-	-	-	-
11	Male	G2/3	HG	pT2	N/A	Positive	-	c.1108G>T	-	Diagnosis	Yes	Positive	c.-124C>T (7.42%)	c.1108G>T (8.71%)	-
12	Female	N/A	N/A	N/A	N/A	Negative	-	-	-	Diagnosis	No	-	-	-	-
13	Male	G1	LG	pTa	N/A	Negative	-	-	-	Diagnosis	Yes	Positive	c.-124C>T (3.58%)	-	-
14	Male	N/A	N/A	N/A	N/A	Negative	-	-	-	Diagnosis	No	-	-	-	-
15	Male	G3	HG	pT1	N/A	Negative	-	-	-	Surveillance	No	-	-	-	-
16	Male	G3	HG	pTa	Negative	Negative	-	-	-	Surveillance	No	-	-	-	-
17	Female	No histology — suspicious on cytology			Suspicious	Negative	-	-	-	Diagnosis	Suspicious	Positive	c.-124C>T (3.74%)	-	-
18	Male	G3	HG	pTa	Suboptimal	Negative	-	-	-	Diagnosis	Yes	Positive	-	c.1111A>T (38.95%)	-
20	Male	G2	LG	pTa	Negative	Negative	-	-	-	Diagnosis	Yes	Positive	c.-124C>T (2.79%)	c.1118A>G (8.25%)	-

Patient	Sex	Grade (WHO 1973)	Grade (WHO 2004)	Stage	Cytology	URM Result	TERTp (NM_198253.3)	FGFR3 (NM_000142.5)	KRAS (NM_033360.4)	URM at Dx or Surveillance	Confirmed Tumour/ Suspicious at URM	OKD Result	TERTp (OncoDEEP)	FGFR3 (OncoDEEP)	KRAS (OncoDEEP)
22	Female	G3	HG	pT1	Negative	Negative	-	-	-	Surveillance	No	Negative	-	-	-

URM, Uromonitor® result; OKD, OncoDEEP® assay result; G, Grade (WHO 1973 classification); LG, low-grade (WHO 2004/2016); HG, high-grade (WHO 2004/2016); TERTp, TERT promoter hotspot mutations (NM_198253.3); FGFR3, fibroblast growth factor receptor 3 (NM_000142.5); KRAS, Kirsten RAS proto-oncogene (NM_033360.4); VAF, variant allele frequency; Dx, first presentation/diagnosis; N/A, not applicable or information not available; –, no mutation detected; Fail, assay technical failure; None, no additional oncogenic variants detected. Patient numbers correspond to those used during study enrolment; patients 19 and 21 were excluded from Cohort C analysis.
